# Bacterial composition in Swedish raw drinking water reveals three major interacting ubiquitous metacommunities

**DOI:** 10.1002/mbo3.1320

**Published:** 2022-09-26

**Authors:** Björn Brindefalk, Harald Brolin, Melle Säve‐Söderbergh, Edvin Karlsson, David Sundell, Per Wikström, Karin Jacobsson, Jonas Toljander, Per Stenberg, Andreas Sjödin, Rikard Dryselius, Mats Forsman, Jon Ahlinder

**Affiliations:** ^1^ CBRN Security and Defence, FOI, Swedish Defence Research Agency Umeå Sweden; ^2^ Department of Molecular and Clinical Medicine, Institute of Medicine, Sahlgrenska Academy University of Gothenburg Gothenburg Sweden; ^3^ Science Division Swedish Food Agency Uppsala Sweden; ^4^ Institute of Environmental Medicine, Karolinska Institutet Stockholm Sweden; ^5^ Department of Ecology and Environmental Science (EMG) Umeå University Umeå Sweden; ^6^ Department of Biomedical Science and Veterinary Public Health Swedish University of Agricultural Sciences Uppsala Sweden

**Keywords:** 16S rRNA, anthropogenic effects, bacterial community analysis, biotic interactions, generalized linear latent variable model

## Abstract

**Background:**

Surface raw water used as a source for drinking water production is a critical resource, sensitive to contamination. We conducted a study on Swedish raw water sources, aiming to identify mutually co‐occurring metacommunities of bacteria, and environmental factors driving such patterns.

**Methods:**

The water sources were different regarding nutrient composition, water quality, and climate characteristics, and displayed various degrees of anthropogenic impact. Water inlet samples were collected at six drinking water treatment plants over 3 years, totaling 230 samples. The bacterial communities of DNA sequenced samples (*n* = 175), obtained by 16S metabarcoding, were analyzed using a joint model for taxa abundance.

**Results:**

Two major groups of well‐defined metacommunities of microorganisms were identified, in addition to a third, less distinct, and taxonomically more diverse group. These three metacommunities showed various associations to the measured environmental data. Predictions for the well‐defined metacommunities revealed differing sets of favored metabolic pathways and life strategies. In one community, taxa with methanogenic metabolism were common, while a second community was dominated by taxa with carbohydrate and lipid‐focused metabolism.

**Conclusion:**

The identification of ubiquitous persistent co‐occurring bacterial metacommunities in freshwater habitats could potentially facilitate microbial source tracking analysis of contamination issues in freshwater sources.

## Graphical abstract

The composition of microbial communities was investigated at six Swedish freshwater sites used as a source of raw drinking water over several years. By model‐based methodology, a ubiquitous structure in the bacterial composition was found where three different metacommunities of co‐occurring taxa were repeatedly identified: these metacommunities differed in response to environmental factors and in metabolic capability, providing unique insights that biotic interactions might be an important driver of microbial occurrence in waters of boreal temperate regions. The findings have the potential to increase knowledge of microbial ecology in these biomes and as an extension provide improvements in drinking water sources monitoring methodology.

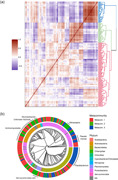

## 1 INTRODUCTION

Access to clean water is of global importance to public health and a key factor in maintaining a well‐functioning society. Future challenges—arising due to increased urbanization and climate change—are expected to reduce freshwater quality, resulting in increased particle and nutrient load, but also fecal pollution (Arnell et al., 2015; Howard et al., 2016; Vörösmarty et al., 2000). Due to better, more readily available sequencing capabilities at a lower cost, several recent studies have been able to assess the anthropogenic impact on bacterial composition in watersheds (Hägglund et al., 2018; Llirós et al., 2014; Newton & McLellan, 2015; Shen et al., 2019). These studies indicate that anthropogenic actions have a clear impact on bacterial diversity along eutrophic‐oligotrophic gradients. Eutrophication can disturb the microbial community composition, altering the carbon and nutrient cycling and, as a result, the entire aquatic ecosystem (Kiersztyn et al., 2019; Newton & McLellan, 2015; Nyirabuhoro et al., 2020; Zeng et al., 2019). Regarding the impact of anthropogenic activity on microbial diversity and function, few long‐term longitudinal studies have been conducted, emphasizing the need for increased knowledge of seasonal and interannual changes in biodiversity at the community level. Due to the high turnover rate of most prokaryotes, as compared to larger organisms, long‐term trends in microbial communities are of particular interest, as these communities have the potential to change more over time, thus resulting in a faster response to anthropogenically induced perturbations.

Most studies of bacterial community composition in boreal lakes, which are of specific importance as sources of drinking water in temperate regions, have focused on inferring factors shaping bacterial community structure and possible correlations within the communities. These studies have described waters in the Nordic countries (Eiler & Bertilsson, 2007; Eiler et al., 2012, 2013; Peura et al., 2012) and in other similar environments, such as freshwater bog lakes in the northern United States (Linz et al., 2017) and boreal lakes in Québec, Canada (Cheaib et al., 2018; Niño‐García et al., 2017) as well as lakes and ponds across Europe (Bock et al., 2020). Eiler et al. (2012) examined the temporal dynamics of bacterioplankton communities in Lake Erken situated in eastern Sweden and found temporal trajectories over annual cycles and complex inter‐dependencies within communities which point toward the importance of biotic interactions (such as direct competition/mutualism as well as less direct interaction) for shaping community structure.

However, as pointed out by Langenheder and Lindström (2019), a limitation of the aforementioned studies is that they have either focused on a long time series for a single lake or single/few time points across many lakes. Therefore, further studies of longitudinal data collected in multiple lakes are warranted to understand the complex associations of diversity, interactions within and between communities, and the influence of environmental and anthropogenic factors, to understand the general governing principles of microbial composition and ecology. From a water safety management point of view, large‐scale longitudinal studies are required to better define the variability in the community as true perturbations due to external factors will be more easily identifiable and discernible from natural fluctuations by employing the results of such studies. Discriminating natural fluctuations from external anthropogenic changes in the composition will also greatly improve microbial source tracking performances (Hägglund et al., 2018; Read et al., 2015).

In the present study, six Swedish raw water sources were sampled for 3 years. The water sources represent diverse environments including both anthropogenically affected and more undisturbed waters. The study aimed to describe the composition and inferred metabolic capabilities of the microbial communities across a timescale of several years to assess if factors linked to anthropogenic perturbation may shape the diversity, interactions, and capabilities of the present microbes and if a difference in these patterns between affected and pristine waters could be observed. The longitudinal design of the study gave us a unique opportunity to infer fine‐scale biotic interactions while simultaneously accounting for other sources of variation induced by anthropogenic disturbance and meteorological and location factors.

## 2 EXPERIMENTAL PROCEDURES

### 2.1 Selection of sampling locations—chemical and physical properties

**Figure 1:**
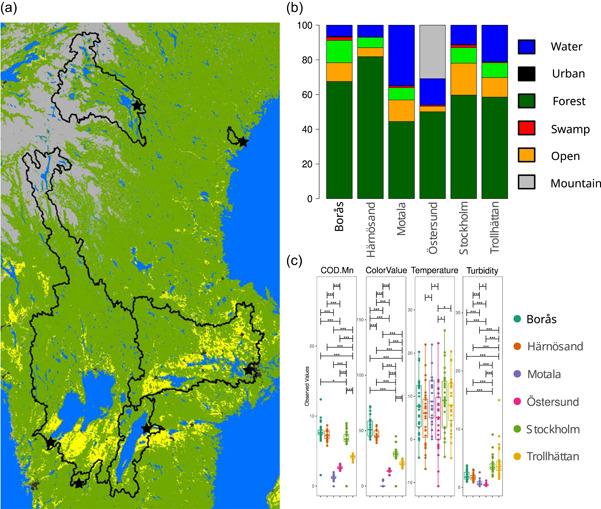
(a) Watersheds in Sweden with locations of the selected drinking water treatment plants (DWTPs). Black outlines show the extent of the catchment area of watersheds for each of the six DWTPs. (b) Land use fraction of the watersheds for the six DWTPs with dark green = forest, yellow = farmland, light green = open land, red = swamps and wetlands, black = urban terrain, blue = water, and grey = other (mainly mountains). (c) Environmental parameters for the six selected DWTPs for the study period. Brackets show a significant difference between locations as determined by the analysis of variance test (*p = 0.05; **p = 0.01; ***p = 0.001).

The sampling locations were selected from a set of 200 surface raw drinking water sources monitored from 2000 to 2011. During the monitoring period COD‐Mn (Method Fd. SS 02 81 18), color value, turbidity, and the number of cultivable microorganisms at 22°C (ISO6222) were measured. Of the parameters selected for inclusion in the modeling, wind, temperature, and amount of rain represent meteorological factors, while turbidity and color values refer to the physical properties of the water. “Cultivable colony forming units (CFU)” was included as a direct biological factor. Additionally, average air temperature and precipitation were calculated for the week before each sampling. From the initial 200 sampling locations, a subset of six water sources was selected for more in‐depth study, representing both lakes and flowing waters from the south and middle of Sweden and ranging in size from smaller lakes to the largest river by average flow rate in Sweden. The selected freshwater sources were situated in the areas of Stockholm, Östersund, Motala, Borås, Härnösand (lakes), and Trollhättan (river). The watersheds providing the source of inflow for the six sampling locations represent a diverse complement of land use, including almost pristine wood‐land boreal lakes and heavily anthropogenically affected sources influenced by extensive farming, urban areas, and industrial development (Figure 1a,b and Table 1). To characterize the sites, an additional set of more extensive water quality indicators were measured during a longer period, including coliforms, *Escherichia coli*, enterococci, *Clostridium perfringens*, and somatic coliphages (Hägglund et al., 2018).

**Table 1:** Area in square kilometers and percentage of land area classification for each watershed

Location	Size (km^2^)	Water	Urban	Forest	Wetland	Open	Mountain
Borås	455	5.42	2.30	56.47	0.00	35.81	0.00
Härnösand	283	3.74	0.20	84.62	0.00	11.44	0.00
Motala	6561	34.16	1.16	39.68	1.13	23.88	0.00
Östersund	11,401	12.81	0.26	56.67	5.85	3.77	20.64
Stockholm	22,550	10.40	1.98	53.87	0.45	33.31	0.00
Trollhättan	40,055	21.03	0.49	55.01	2.13	20.97	0.38

### 2.2 Sample collection, DNA extraction, library construction, and sequencing

Raw water samples were collected from inlets at six drinking water treatment plants (DWTP). Between September 2013 and August 2015, a total of 230 raw water samples were collected of which 175 were selected for DNA sequencing. DNA was amplified with bacteria/archaeal primers 515 F/806 R specific for the hyper‐variable V4 region of the 16S rRNA gene (Caporaso et al., 2012). The obtained polymerase chain reaction (PCR) amplicons were sequenced on an Illumina MiSeq platform using a combination of 300‐ and 500‐bp paired‐end kits. Sampling procedures, DNA extraction, and sequencing are described and performed in Hägglund et al. (2018).

### 2.3 Bioinformatic analysis of sequencing data

The sequences were demultiplexed using the deML software (Renaud et al., 2015). The reads were quality filtered and denoised using DADA2 (Callahan et al., 2016, 2017). The “filterAndTrim,” “learnErrors,” and “dada” functions were run on each of the eight amplicon sequencing data sets to train the error model specifically for each set so that heterogeneity in sequencing runs was accounted for. Read length truncation parameters were decided based on the Phred quality scores plot for each sequencing run and varied between 140 and 180 for forward reads and between 120 and 135 for reverse reads. The maxEE parameter was set to default (i.e., equal to 2.0). Reads were truncated at the first instance of a quality score less than or equal to 11 (i.e., truncQ). Chimeras were removed based on the consensus method in the function removeBimeraDenovo. After the quality filtering, 68.7% of the reads were retained. As two different Illumina MiSeq reagent kits were used through the sequencing of the samples, the reads had to be truncated to match the kit with the shortest read‐length, although the final sequence lengths were sufficiently long to overlap the paired‐end reads successfully (i.e., ranging between 15 and 60 bp overlap). Then the data were filtered as follows: reads shorter or longer than 2 × SD (242, 263 bp) were removed, and amplicon sequence variants (ASVs) unclassified at the Kingdom level were also removed. This pruning reduced the number of ASVs from 40,175 to 40,012. Subsequent removal of eukaryotic (mitochondria and chloroplast) sequences decreased the number of ASVs to 39,081 (and the total number of reads to 20,269,784). The maximum and minimum number of read counts were 433,918 and 27,958, respectively, with a mean read count of 124,865 (Table A3).

### 2.4 Statistical analysis and visualization

The clustering of water samples and sites into two partitions, corresponding to anthropogenically disturbed and pristine environments, was performed on the extended set of 230 samples using nine water quality indicators: CODMn (Chemical Oxygen Demand), color value, turbidity, coliforms, *E. coli*, enterococci, cultivable bacteria at 22°C, *C. perfringens*, and somatic coliphages. The samples were assigned to either environment using both k‐means unsupervised clustering and principal component analysis (PCA) in R on scaled variables (i.e., all indicator data were transformed so that the mean was zero and the variation was one) with default parameter values in both analyses. At the site level, a 50% cutoff (of a proportion of samples) determined membership in the respective environment. The taxonomic composition of the bacterial communities was analyzed and visualized using the phyloseq R package (McMurdie & Holmes, 2013). Visualization and statistical computation of environmental data and alpha diversity were performed using the R package (microbiomeseq v. 0.1 https://github.com/umerijaz/microbiomeSeq/) and the Phylosmith R package (phylosmith v. 1.0.6 https://schuyler-smith.github.io/phylosmith/). To infer differences in mean values between distributions of both environmental data and alpha diversity per site, analysis of variance was performed with comparisons where *p* values lower than 0.05 were indicated. PCA was performed using center‐log transformed values to visualize differences between communities using the MicroViz R‐package (Barnett et al., 2021). To infer a phylogenetic tree for the top 200 (i.e., the 200 most numerous) ASVs, RaxML version 8.2.X (Stamatakis, 2014) with standard settings and the GTRCAT approximation of nucleotide substitution rate heterogeneity was used. Phylogenetic trees were visualized using the ggtree package (Yu et al., 2017) in R. Heatmaps were visualized using the ggheatmap function in the R package heatmaply (Galili et al., 2018). Detection of a phylogenetic signal for metacommunity distribution was performed with the delta‐statistic method presented by Borges et al. (2019), using default settings of the delta function in R and 1000 permutations to create the distribution of delta under the null hypothesis of no signal between the trait and the phylogeny.

### 2.5 Multivariate generalized linear modeling of interactions between taxa

To investigate the effect of environmental predictors on the communities and biotic interactions within, a multivariate generalized linear latent variable model (GLLVM) was fitted to the community data through the gllvm R package (Niku et al., 2019). By modeling the response of abundance to predictors jointly with the correlation across taxa, we have the possibility of teasing the two apart by explicitly modeling the correlation structure via latent variables. In doing so, we can both estimate the effects of the environmental predictors and residual correlations jointly (Caradima et al., 2019; Ovaskainen et al., 2017; Warton et al., 2015). The ASV abundance matrix was set as a response variable and rescaled and centered average air temperature, turbidity, COD‐Mn, and Color values were set as continuous predictors and water plant location and season of sampling were used as group‐level predictors according to:

(1) $$g\left(m_{ijkl}\right)=\beta_{0ij}x_{0i}+\beta_{1ij}x_{1i}+\beta_{2ij}x_{2i}+\beta_{3ij}x_{3i}+\beta_{4ij}x_{4i}+\beta_{5ijk}+\beta_{6ijl}+z_{jk}\lambda_{k},$$

g() is the log link function defining the mean of the linear function of predictors, *m_ij_* is the *j*th ASV abundance in sample *i* (*i* = 1…163), *β*_*0i*_ is the effect of the total sample sequence abundance for sample *i* on ASV *j* (*j* = 1…200, as only the top ASVs in terms of abundance were included), *β*_1*i*_ is the effect of the average air temperature (1 week before the sampling) for sample *i* on ASV *j*, *β*_2*i*_ is the effect of turbidity for sample *i* on ASV *j*, *β*_3*i*_ is the effect of COD‐Mn for sample *i* on asv *j*, *β*_4*i*_ is the effect of color values for sample *i* on ASV *j*, *β*_5*jk*_ is the effect of the sampling site *k* (*k* = 1…6) on ASV *j*, and *β*_6*jl*_ is the effect of season *l* (*l* = 1…4) on the *j*th ASV. The latent (unobserved) variables *z*_*jk*_ were included to explain the residual covariance structure. Further information on the GLLVM analysis is supplied in Appendix A (see Materials and Methods section). To check for co‐linearity, correlations between predictors were estimated using the base cor function in R: results are provided in Appendix A and Figure A13.

### 2.6 Reconstruction of community metabolic pathways

To reconstruct the metabolic capability from ASV data of the three defined metacommunities obtained in the GLLVM analysis, PICRUSt2 v.2.3.0‐b (Douglas et al., 2020) was run using a standard setting on un‐rarified ASV‐table data of the top 200 most abundant taxa and representative sequences corresponding to each ASV. The DESeq. 2 (Love et al., 2014) R package was used to detect differentially abundant KEGG (Kyoto Encyclopedia of Genes and Genomes) orthologues and BioCyc pathways. To deal with zeroes in the data set a pseudocount of +1 was added to each data point, and the results were subjected to variance stabilizing transformation to reduce skew (Anders & Huber, 2010; Huber et al., 2003; Tibshirani, 1988).

### 2.7 Analysis of land usage in catchment areas

The catchment areas for each sample point were based on data obtained from the Swedish Meteorological and Hydrological Institute. Land cover or land use for each catchment area was estimated using CORINE Land Cover data from Copernicus (https://land.copernicus.eu/pan-european/corine-land-cover). The land cover types were pooled into six categories, that is, water surface, open ground, swamp, mountain, forest, and urbanized area.

## 3 RESULTS

To perform an in‐depth study of the difference in water properties at the selected sites, representing a wide diversity in the catchment area and land use (Table 1 and Figure 1a,b), a total of 230 samples were collected between 2013 and 2015. All samples were analyzed for water quality (i.e., CODMn, color value, turbidity, cultivable microorganisms at 22°C, *E. coli*, *C. perfringens*, enterococci, coliforms, and somatic coliphages), and a subset of 175 samples was subjected to 16S amplicon sequencing (Hägglund et al., 2018) with a broader representation of biological, meteorological, and physical properties included (Figure 1c). The various factors showed a differing degree of variability, both between and within sample sites, with color value displaying the highest between site variability and temperature showing the greatest within‐site differences. Both Stockholm and Trollhättan had significantly higher mean turbidity than the other sites, while Borås and Härnösand showed significantly higher mean color values. Trollhättan samples also showed higher levels of cultivable bacteria at 22°C than at the other sites.

**Figure A1:**
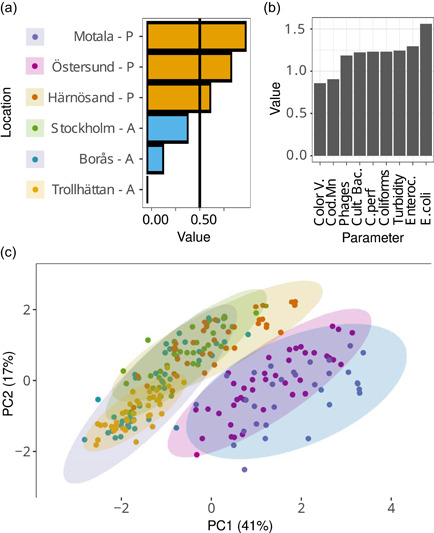
Clustering of 230 water samples into two partitions based on water quality indicator variables, highlighted by the proportion of samples assigned to the undisturbed partition using k‐means clustering (a) and the average difference between partitions of normalized indicator variable abundance based on the clustering result (b), as well as with a PCA plot of PC1 and PC2 (c). Character adjacent to DWTP name (A/P) indicates if a location was classified as Anthropogenically affected or Pristine. DWTP, drinking water treatment plant; PCA, principal component analysis.

**Figure A2:**
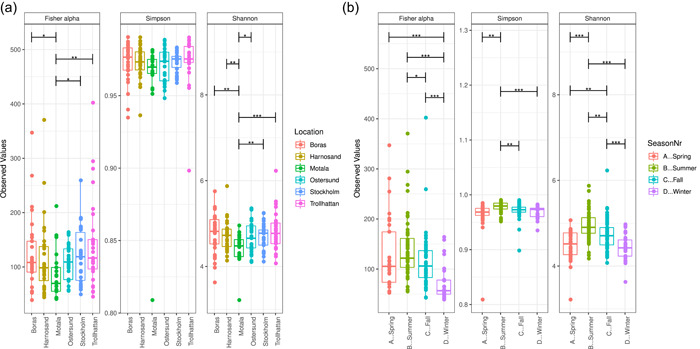
Diversity plots highlighting differences in alpha diversity, grouped according to locale (a) and season (b). Brackets show significant differences in groups as determined by the analysis of variance test with * signifying *p* = 0.05, **p = 0.01, and ***p = 0.001. Before analysis sequences corresponding to mitochondria and chloroplasts were removed, otherwise unfiltered (but rarefied to even depth) data was used.

The water quality data of all 230 samples were assigned into two partitions (anthropogenic affected and unaffected/pristine environments), for which 94%, 80%, and 60% of all samples from Motala, Östersund, and Härnösand, respectively, were assigned to the unaffected group, while 100%, 85% and 62% of Trollhättan, Borås, and Stockholm samples were assigned to the anthropogenic affected group (Figure A1a). The most important indicator in the partition was *E. coli* with an average difference between the partitions of 1.56, while CODMn and color value were the least discriminative with an average difference of 0.90 and 0.86. All the other indicators were intermediate discriminative with a difference of 1.23–1.29 (Figure A1b). This resulting partition was supported by the PCA (Figure A1c), as the first PC with the highest proportion of explained variation in indicator data (41%), showed a similar partitioning of the sites, while the other components explained considerably less (17%, 10%, and 10% for PC2, PC3, and PC4, respectively). When comparing the overall bacterial diversity of the raw water at the sites (Figure A1), as measured by a few standard diversity indices, Motala and Härnösand displayed the lowest diversity, although the difference was only significant for Motala. The low diversity of Härnösand is likely because this water represents a biologically divergent environment compared to the other sampled locations, with a higher amount of humus typical of boreal lakes. Motala on the other hand takes its water from lake Vättern, the second largest lake in Sweden with generally good (i.e., oligotrophic, cold, nutrient‐poor, and oxygen‐rich) water quality indices, reflecting an environment with fewer exploitable microbial niches. Waters from other larger sources (Stockholm and Östersund, lakes Mälaren and Storsjön, respectively), displayed lower variability due to the effects of much larger surface area and volume leading to slower response times indicative of more stable environments. As expected, diversity peaked in summer and was the lowest in winter (Figure A2).

**Figure 2:**
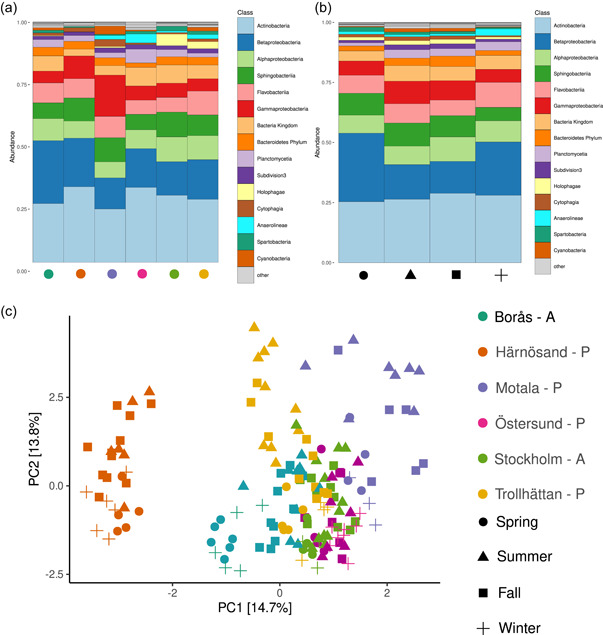
Barplots showing the relative composition at the phylum level of samples for the 200 most common taxa, grouped by location (a) and season (b). (c) Center‐log ratio transformed PCA showing the relative composition of sampling sites for the top 200 taxa. Colors correspond to the sampling location while shapes correspond to the sampling season. Character adjacent to DWTP name (A/P) indicates if a location was classified as Anthropogenically affected or Pristine. DWTP, drinking water treatment plant; PCA, principal component analysis.

**Figure A3:**
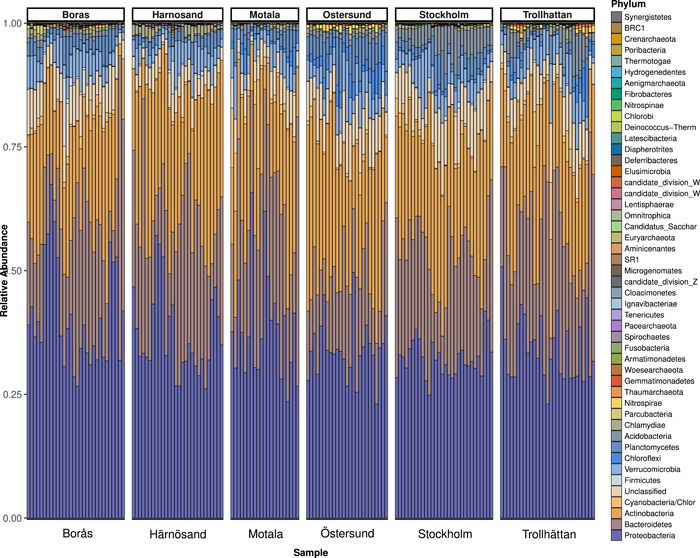
Taxonomic profile of all samples at the phylum level, separated by sampling location

Microbial communities at all sample sites were dominated by three phyla (Figures 2 and A3), common for water sampled from freshwater lakes and in line with previous investigations on Swedish waters (Eiler et al., 2012; Peura et al., 2012). Phyla Actinobacteria, Bacteroidetes, and Proteobacteria (to a large extent comprised of α‐proteobacteria of the order Pelagibacterales belonging to the common freshwater LD12 clade) constituted approximately 80%–90% of all taxa, with variations between sites due to abundance of less common phyla. Actinobacteria were slightly more common at the northern sampling sites of Härnösand and Östersund, while Östersund also displayed an elevated abundance of Chloroflexi compared to the other waters. Of note is that Motala was the only site showing significant numbers of cyanobacteria in some samples, possibly corresponding to annual bloom events (Figure A3).

General trends were also observed for seasonal variation of the top 200 taxa (Figure 2b) in microbial composition, with the actinobacterial fraction showing a peak in fall, and the proteobacterial fraction peaking in the winter/spring season. Furthermore, larger fractions of Planctomycetes and Chloroflexi were present during the fall/winter season, while Actinobacteria and Verrucomicrobia mainly appear to be associated with the summer/fall period.

Unclassified sequences were present in all waters, to a varying degree both geographically and temporally, pointing to a still unexplored diversity present in Swedish freshwaters.

**Figure 3:**
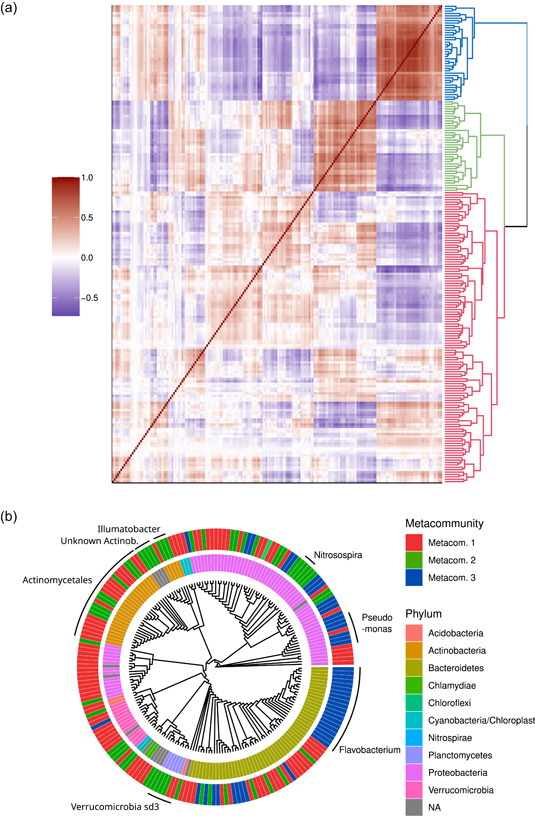
(a) Heatmap of correlations between amplicon sequence variants (ASVs) and clustering dendrogram with the three metacommunities highlighted as separate colors, red, green, and blue for metacommunities 1, 2, and 3, respectively. The legend shows the correlation interval. (b) Cladogram of the top 200 ASVs and their metacommunity and taxonomic assignments at the phylum level. Color coding for metacommunity association as per description for (a).

A PCA (center‐log transformed data) analysis of the top 200 taxa representing the highest relative abundance (Figure 3c) revealed Härnösand (and to a lesser degree Motala) as the most divergent locale in terms of which taxa are most relatively abundant, while Stockholm represents the most median sampling site.

**Figure A4:**
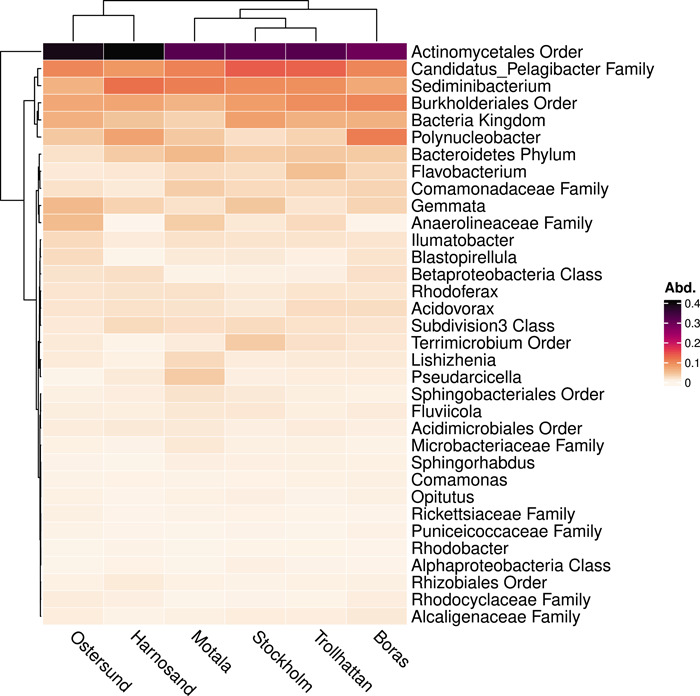
Heatmap showing the core taxa present in at least 90% of the samples. In cases where no classification on the Genus level was present, the lowest identified taxonomic level is given after the taxa name.

Overall, the relative abundance of taxa present in at least 90% of all samples was comprised of a number of known generalist freshwater clades showing a fairly even distribution in all samples, the most numerous clades being various Actinomycetales and other Actinobacteria, although no single taxon exceeded approx. 0.5% of the total fraction in any individual sample (Figure A4). Furthermore, several well‐known fresh‐water bacteria were detected in lower fractions but showed little variation between samples such as the wide‐spread alpha‐proteobacterial LD12 clade, *Sediminibacterium, Polynucleobacter*, and other Burkholderiales, unidentified members of phylum Bacteroidetes and subdivision 3 of phylum Verrucomicrobia, and several members of the beta‐proteobacterial family Comamonadaceae. Other notable taxa displayed relatively high abundance in a selection of samples but had a spottier distribution, such as genera *Fluviicola, Terrimicrobium, Blastopirellula, Anaerolineaceae, Rhodoferax, Acidovorax*, members of families Rhodocydaceae and Alcaligenaceae, in addition to genera *Flavobacterium* and *Sphingorhabdus*.

To summarize the general diversity analysis of the raw water assemblages, we have identified a complex pattern of variation between sites, seasons, water quality parameters, and sequence taxonomy. This observed complexity implies that further modeling is warranted to disentangle the different sources of variation.

### 3.1 Model analysis reveals three distinct and abundant metacommunities

**Figure A5:**
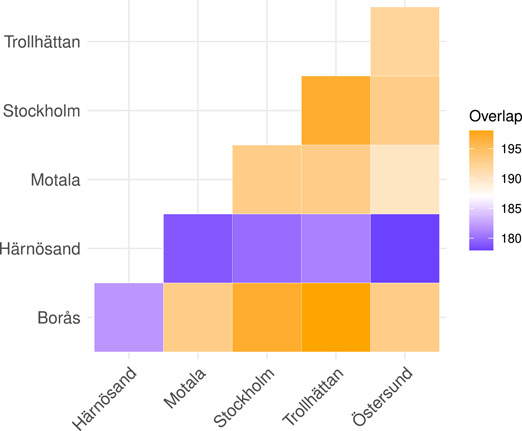
Heatmap showing the amount of shared top 200 abundant amplicon sequence variants between locales. The yellow color indicates a high degree of overlap while blue indicates less overlap.

**Figure A6:**
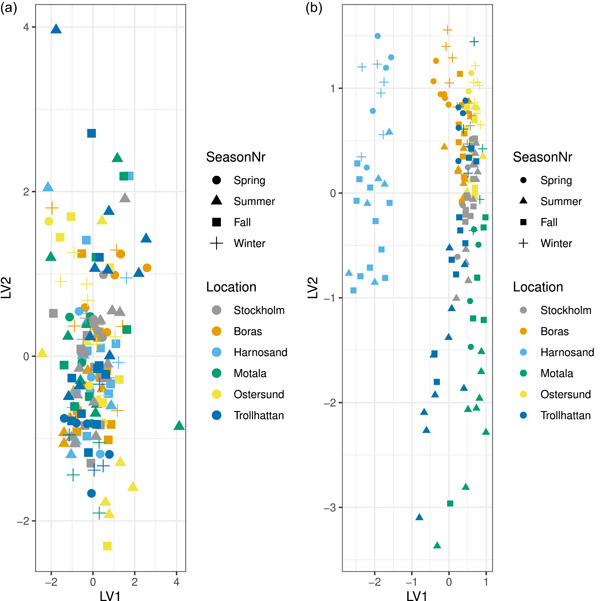
(a) Model‐based ordination with all predictors included in the model shown as latent variable one and two‐parameter space (LV1 and LV2), and (b) ordination without any predictors in the model for LV1 and LV2 (i.e., the null model). For illustration, both site and season factors are highlighted with colors and shapes respectively. The included predictors of the full model explained 41% of the total variation in amplicon sequence variant abundance.

**Figure A7:**
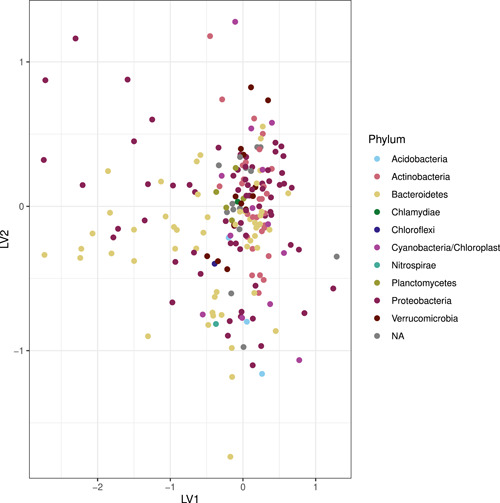
Inferred factor loadings of the GLLVM model of the top 200 ASVs where their respective phylum assignments are highlighted in colors, with the first two dimensions of the latent variables (LV) shown. ASV, amplicon sequence variant; GLLVM, generalized linear latent variable model.

To determine if bacterial assemblages responded to habitat characteristics and displayed signs of interactions among ASVs, a GLLVM was fitted to the ASV occurrence data, with turbidity, color value, air temperature, and COD‐Mn included as covariates and location and season as factor level predictors in the model. Only the top 200 ASVs in terms of total abundance were included, with a high degree of ASVs being shared (i.e., present in at least 90% of samples and with an abundance of at least 0.1% of reads) between locations (Figure A5). The selection of the most common taxa and the exclusion of comparably more rare taxa were performed as these likely represent biologically important species and are less sensitive to both, noise in the data (i.e., close to detection limits, PCR induced bias, database incompleteness) and problems with compositionality inherent to all sequencing analyses with technical limits to sequencing depth (Alteio et al., 2021). All inferred associations with 95% confidence intervals are provided in Table S1 available at 10.5281/zenodo.7066483. When including all predictors in the model, 41% of the total variation was accounted for in the analysis, which better allows us to draw conclusions from the inferred ASV correlations after adjusting for the predictors (Figure A6). By inspecting the inferred factor loadings of the model, the ASVs that explain most variation in ASV abundance were assigned to the phyla Proteobacteria and Bacteroidetes (Figure A7). A variable importance analysis was performed where the geographic location effect was found to be most influential (of the included predictors) on composition (Table A1).

**Figure A8:**
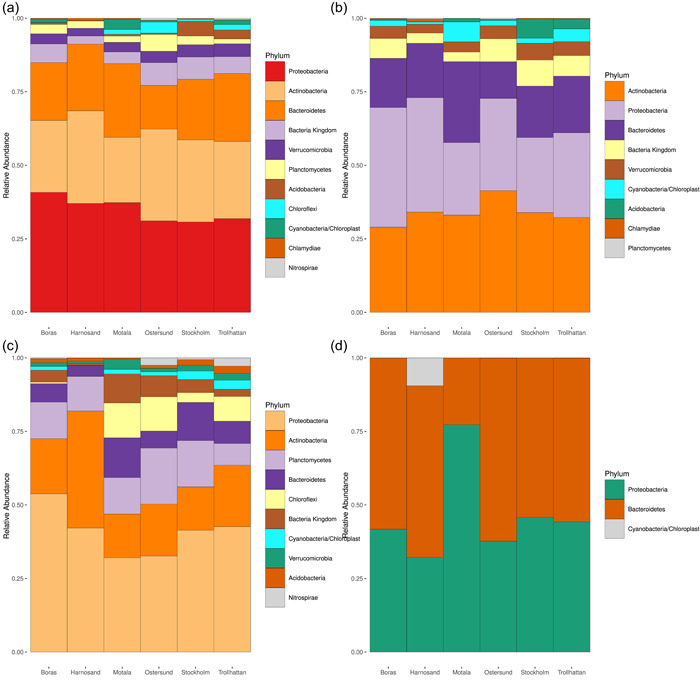
Taxonomic composition at the phylum phylogenetic level of (a) top 200 taxa, (b) clade 1 metacommunity, (c) clade 2 metacommunity, and (d) clade 3 metacommunity

**Figure A9:**
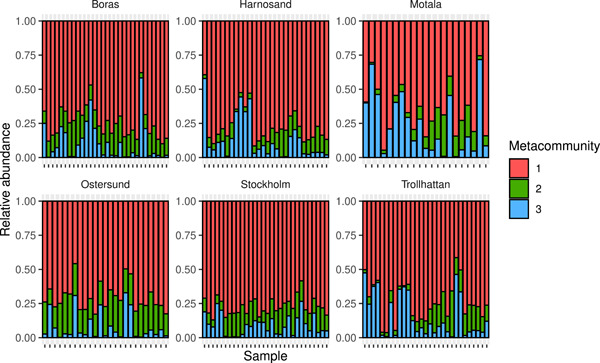
Barplots of the relative abundance of the top 200 amplicon sequence variants assigned to each metacommunity plotted per sampling location

Potential biotic interactions based on the results of the GLLVM analysis were analyzed by assessing the estimated correlations across ASVs. Three distinct metacommunities were identified using hierarchical clustering (Figure 3a). The first metacommunity did not exhibit any clear pattern on average correlation within (*r*_1_ = 0.056 [0.196]) and between communities (results not shown), while the second and third metacommunities displayed strong positive within community correlation (mutualism; *r*_2_ = 0.330 [0.201], *r*_3_ = 0.499 [0.205]) and negative between community correlation (*r*_2,3_ = −0.249 [0.174]). The three metacommunities consisted of members with different taxonomic affiliations at the phylum level (Figures 2b and A8). Metacommunity 1 and 2 were highly diverse with most phyla present in the data set represented, although in both cases Actinobacteria and Proteobacteria were dominant, with Bacteroidetes representing a significant fraction of metacommunity 1 but mostly present in lower amounts in metacommunity 2. Metacommunity 3 was almost exclusively composed of Bacteroidetes and Proteobacteria, in contrast to the other two communities, and showed a lower diversity at the phylum level. In contrast to community 1, which mostly mirrored the general diversity in the data set, communities 2 and 3 comprised taxa associated with specific conditions and consisted of mutually exclusionary and unique organisms. Worth noting is that members from the same metacommunity tended to cluster together in the phylogeny. This clustering distribution of metacommunities in the phylogeny was tested using delta‐statistic, with an obtained *δ*_m_ = 3.28 compared to *δ*_0_ = 2.12 (0.63) of the null distribution (*p* = 0.000), which implies the presence of a phylogenetic signal (i.e., the ecological similarity between taxa is related to phylogenetic relatedness), showing that specific life‐strategies adopted by taxa is governed to an extent by shared evolutionary history although this can vary between taxa even if comparing on the same taxonomic level. The spatiotemporal trends of the three metacommunities were accessed by investigating their relative contribution to each assemblage (Figure A9). On average, metacommunity 1 was the most abundant. Both, temporal dependency on metacommunity compositions and side effects were observed, where metacommunity 2 was most abundant (on average) in Östersund and Borås (average frequencies of 21.2% and 15.0%, respectively) while metacommunity 3 was most abundant in Motala and Trollhättan (24.5% and 16.0%, respectively).

**Figure 4:**
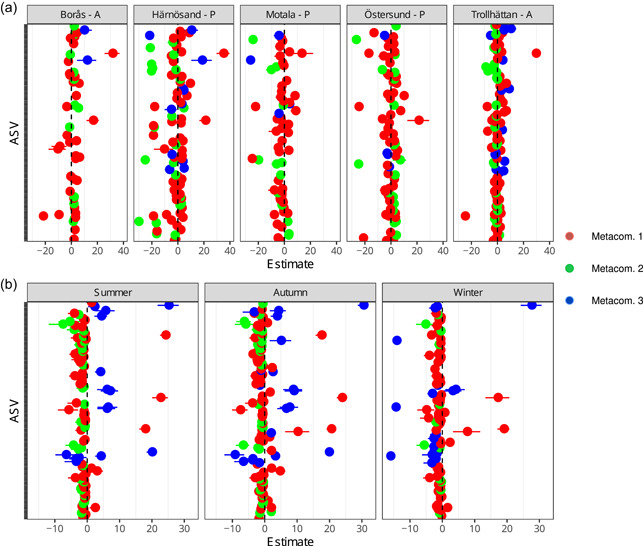
The estimated coefficient of corresponding ASVs associated with: (a) site effects where Stockholm DWTP is set as a reference and (b) season effects where spring is set as a reference level. The estimated mean value is shown as a point with 95% CI as lines around the point. Only coefficients with intervals not including zero effect size are shown here. The colors of the coefficients, red, green, and blue correspond to metacommunities 1, 2, and 3, respectively. Character adjacent to DWTP name (A/P) indicates if a location was classified as Anthropogenically affected or Pristine. AVS, amplicon sequence variant; CI, confidence interval; DWTP, drinking water treatment plant.

**Figure 5:**
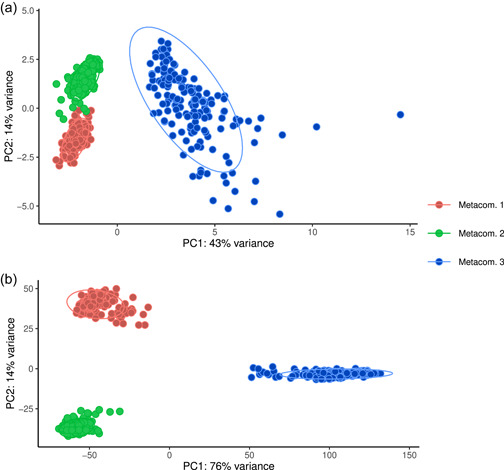
PCoA plot of differential abundance values for (a) BioCyc metabolic pathways and (b) KEGG orthologues of the three metacommunities. Color coding of data points is according to results from model analysis designation to the three metacommunities where red, green, and blue correspond to metacommunities 1, 2, and 3, respectively. Ellipses correspond to 95% confidence intervals. KEGG, Kyoto Encyclopedia of Genes and Genomes; PCoA, principal coordinate analysis.

**Figure A10:**
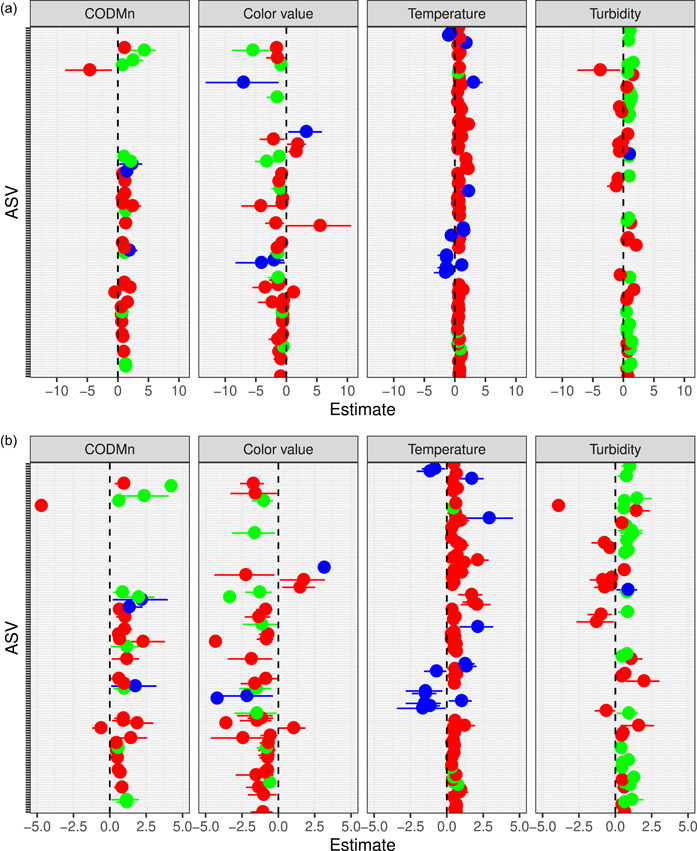
(a) The estimated coefficient of corresponding ASVs associated with environmental predictors included in the model analysis. The estimated mean value is shown as a point with 95% CI as lines around the point. Only coefficients with intervals not including zero effect are shown here. The colors of the coefficients, red, green, and blue correspond to metacommunities 1, 2, and 3, respectively. (b) The estimated coefficient of corresponding ASVs associated with environmental predictors included in the model analysis, where only small but significant effects are shown. The estimated mean value is shown as a point with 95% CI as lines around the point. The colors of the coefficients, red, green, and blue correspond to metacommunities 1, 2, and 3, respectively. ASV, amplicon sequence variant; CI, confidence interval.

**Figure A11:**
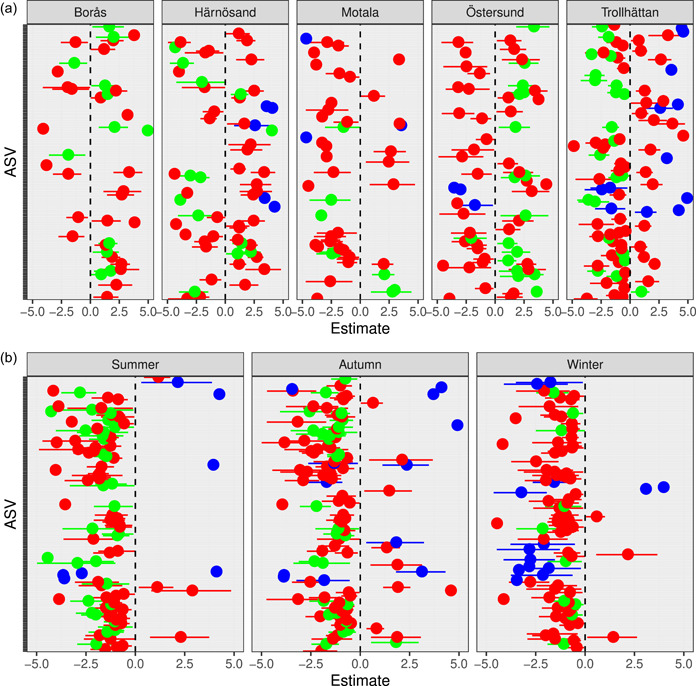
The estimated coefficient of small, but significant, effects corresponding ASVs associated with: (a) site effects where Stockholm DWTP is set as a reference and (b) season effects where spring is set as a reference level. The estimated mean value is shown as a point with 95% CI as lines around the point. The colors of the coefficients, red, green, and blue correspond to metacommunities 1, 2, and 3, respectively. ASV, amplicon sequence variant; CI, confidence interval; DWTP, drinking water treatment plant.

**Figure A13:**
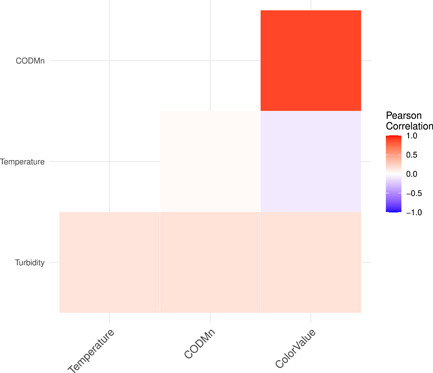
Heatmap showing inferred pair‐wise Pearson correlations between continuous predictor variables included in the full generalized linear latent variable model

In the GLLVM analysis, associations between the taxa and environmental, seasonal, geographic, and water quality variables were inferred: all significant associations are highlighted in Figures 4, A10, and A11 while the highest positive and negative associations for each predictor are shown in Table A2. Turbidity resulted in the most positive significant associations, with members from metacommunity 2 over‐represented. Additionally, the air temperature resulted in many positive associations. Most taxa showed a negative response to higher color values, except for two representatives assigned to metacommunity 1 (Alkaligenaceae and *Polynucleobacter*). For the season predictor, most ASVs with a significant association between spring and any other season were over‐represented in the spring assemblages (i.e., a negative effect size value in Figure 5a). When contrasting summer against spring factor levels, a greater number of taxa belonging to metacommunities 1 and 2 were showing a moderate decrease during summer, while members of metacommunity 3 showed an over‐representation during summer. The overall fall season response was similar to the summer response, while the winter season resulted in most taxa showing an under‐representation. Investigations of the impact of the site generally revealed that ASVs assigned to metacommunities 1 and 2 showed a higher number of associations than ASVs assigned to metacommunity 3, further reinforcing that metacommunity three members consist of generalist bacteria (Figure A8d). In general, members of the same metacommunity showed joint preference, albeit with some exceptions.

To summarize the results of the GLLVM analysis, three distinct metacommunities were identified in the top 200 ASV co‐occurrence data. These metacommunities showed a distinct community structure and associations to the measured environmental data. Members of metacommunity 1 showed a positive estimated response to air temperature, while for metacommunity 2, many positive associations between ASV co‐occurrence and turbidity were inferred. Both site and season factors resulted in many differentially abundant ASVs indicating the presence of a spatiotemporal effect on Swedish raw water assemblages.

### 3.2 The predicted function of metacommunal metabolic potential reveals specific adaptations

To further investigate these metacommunities, an analysis of predicted metabolic functions was undertaken so that differences in function pertaining to the ecological roles fulfilled by the communities could be investigated.

Reconstruction of the theoretical metabolic capability of the top 200 most abundant taxa by use of the PICRUSt2 software for functional inference was performed, followed by differential abundance analysis of the resulting KEGG orthologues and METACyc pathways for each sample site and taxa. Results showed that the three metacommunities inferred from the model analysis differ in favored orthologue/pathway abundance, as shown by a PCA plot of the relative abundances of metabolic features as shown in Figure 5.

Hierarchical clustering of the metabolic capability of the three metacommunities revealed that they can clearly be distinguished on metabolic capability alone, as illustrated in Figures 5a and A12a–d. Intriguing details emerge showing that metacommunity 1, when considering the more abundantly observed orthologues/pathways, appears to favor a diverse metabolism with no specific focus, reflecting the diverse composition of this metacommunity with constituting taxa from many different bacterial phyla. The greatest number of highly abundantly observed metabolic associated orthologues belong to carbohydrate metabolism, something shared with both other metacommunities. Metacommunity 2, on the contrary, showed an increased number of orthologues involved in methane metabolism and other predominantly anaerobic processes, and to a lesser degree amino acid and lipid metabolism. Finally, metacommunity 3 showed the highest amount of increased abundance of carbohydrate metabolism orthologues, in conjunction with an increase of lipid metabolism orthologues.

## 4 DISCUSSION

In this multisite longitudinal study of bacterial communities in Swedish raw waters, with a selection of environmental and meteorological data, degree of land usage, and physical and biological properties of the waters sampled during the study period, we identified several (key) insights. (1) Overall, the total bacterial diversity showed a clear seasonal pattern, with the highest diversity observed in summer. The studied raw water sources were diverse and showed distinct microbial communities separated by geographical location. (2) Nevertheless, we identified a stable, albeit numerically not dominant, set of core taxa (constituting approx. 0.5% of the total amplicon‐determined microbiome). (3) Physical and chemical water parameters commonly measured do not necessarily show a correlation with microbial community composition, suggesting either those unknown parameters not measured may have been responsible for divergent microbiome composition, or that interactions between taxa explained a much larger portion of the variation in composition. (4) The investigated aquatic microbial communities, no matter their geographic origin, contained clearly defined subcommunities, constituting three distinct metacommunities that were identified in the top 200 ASV co‐occurrence data. (5) These metacommunities showed a distinct community structure and associations to the measured environmental data. (6) Finally, the metabolic pathway characteristics of the metacommunities were reconstructed by visualizing the reconstructed abundance, and the observed differences indicate as to which factors determine the observed patterns.

### 4.1 A stable set of core taxa

The different sampling locations presented here represent a variety of trophic conditions and thus a diverse mixture of environments ranging from affected by anthropogenic action (i.e., a greater extent of farm and urban land use) to much less exposed areas. While the sampling locations fall on a gradient, they were divided into two groups for ease of interpretation, here called A (for anthropogenically affected) and P (pristine) as proposed by Numberger et al. (2020). Yet, the detected core set of taxa represents a comparatively small part of the whole community, with taxa present everywhere constituting under 0.5% of the total amount. Given the extensive number of assemblages sampled across seasons and watersheds with very different water quality traits, this small proportion of the total constituting the core microbial community is not surprising. The core microbiome was dominated by representatives within the families of Rhodobacteraceae, Puniceicoccaceae, Sphingomonadaceae, Opitutaceae, Rickettsiaceae, and Microbacteriaceae, with some of the other representative taxa present only at very low abundances, which suggests that the core microbiome consists of generalists that occur within a stable span in frequency, and that this pattern reflects a life‐strategy more oriented towards changing conditions and an opportunistic lifestyle. Our finding that there exist well‐defined core taxa among the different sampling sites, coupled with the results of others (Llirós et al., 2014; Newton & McLellan, 2015; Numberger et al., 2020), suggest that the observed genera contain species with a mixture of oligotrophic and eutrophic preferred conditions. Thus, a plausible conclusion is that within‐genus lifestyle specialization is actively shaping the community composition of freshwater in anthropogenic affected areas.

### 4.2 Noncorrelation of physical and chemical water parameters with microbial community composition

Interestingly, the measured water quality/meteorological variables included in the GLLVM analysis (here CODMn, turbidity, air temperature, and color value) explained a relatively small portion of the total variation in community composition. Thus, the geographical and/or water quality differences at the sampling locations influenced the community composition more heavily in our study area. Spatial effects on composition have been documented on both global and regional scales (Eriksson et al., 2022; Ge et al., 2021), as well as eutrophic status and water quality differences (Shen et al., 2019). The portion of the variation not accounted for here might be a result of several factors. First, there might be factors not assessed in our study design, such as the nutrient and metal richness of the water (Carrero‐Colón et al., 2006; Ge et al., 2021; Sun et al., 2012). Second, the measured data included here are only proxies rather than reflecting the true underlying factors forming the actual gradient of parameters influencing the assemblies. Using the water temperature instead of the air temperature near the location is one such example, and the definition of seasons (based on the meteorological definition) is questionable. Third, biotic interactions between microbes might be an important force, which we have included (in conjunction with unaccounted environmental predictors) in the GLLVM analysis as latent variables approximating an unstructured residual term (Niku et al., 2019; Ovaskainen et al., 2017; Warton et al., 2016).

### 4.3 Effect of anthropogenic impact

We found evidence of taxa augmentation, manifested as approximately higher levels of alpha diversity in water sites in higher anthropogenic affected regions with higher levels of fecal indicator bacteria (Group A, Borås, Trollhättan, and Stockholm) than in those in less‐urbanized regions (Group P, Östersund, Härnösand, and Motala, see also Hägglund et al., 2018). In addition, we found differences between group A and P in relative abundances among common bacterial groups (i.e., top 200 ASVs), where *Methyloparacoccus* had decreased representation, and *Pedobacter* and an unknown bacterial genus, placed within the Ilumatobacter clade, had increased representation in the more heavily anthropogenically affected sites. Similar associations of genera correlated to nutrient richness or eutrophication were reported by Yang et al. (2020), who constructed microcosms with eutrophic freshwater lake sediment to investigate the effect of different ammonium dosages on methanotrophic bacteria communities including Methyloparacoccus.

Other studies have reported a correlation of genera within phyla Proteobacteria and Bacteroidetes to increased nutrient levels, which comes with increased anthropogenic activity, both in microcosms and in lake systems (e.g., Andersson et al., 2018; Buelow et al., 2016; Fisher et al., 2015). Newton and McLellan (2015) found an elevated abundance of the genera Limnohabitans, Polynucleobacter, and Rhodobacter in the urbanized site (urban estuary of Milwaukee), while Numberger et al. (2020) found enriched levels of bacterial families with possible association to fecally contaminated water in urban affected lakes in Brandenburg, Germany, such as Bacteroidaceae, Prevotellaceae, Rikenellaceae, Tannerellaceae, and Weeksellaceae, and defined these as an urban bacterial fingerprint.

Taken together, our results and other studies show that anthropogenic action results in an effect on the structure and composition of the bacterial communities although the microbial taxa constituting the anthropogenic or urban signature differ.

### 4.4 Ubiquitous distinct metacommunities

Interactions between different microbes play an important role in aquatic ecosystem functioning, where biotic interactions can impact the community structure. We found a strong concordance between members of two metacommunities in the selection of aquatic ecosystems included in the study, both within and between communities. If two metacommunities display similar abundances of metabolic pathways, it would seem to indicate that either of the two scenarios is correct; (1) The two communities are in direct competition for the same ecological niche and pure chance and temporal variations determine which one is present in a particular freshwater sample or (2) environmental factors that are not accounted for in the set of predictors (which might explain variation in community composition by evolutionary advantageous adaptations by the respective communities not readily detected by the utilized methods) are responsible for the dominance of the particular metacommunity. On the contrary, if the two communities have clearly different capabilities, it would indicate that the larger assemblies of microbes present in these natural environments are adapted to different ways of utilizing the resources present and that the observed differences have their basis in alternative evolutionary paths and adaptations to different lifestyles followed by the metacommunities.

The three distinct metacommunities partitioned from the top 200 abundant ASV were supported by two independent analyses using two data sources: that is, the co‐occurrence data based solely on abundance and the predicted metabolic pathways of the bacterial communities, based on reconstructed metabolism. In other words, we found that the predicted functionality of the bacterial freshwater metacommunity resembles the inferred correlation pattern among ASVs after adjusting for design and water quality parameters.

To assess if members of the metacommunities were associated with water quality turbidity was used as a proxy. The connection between turbidity and other water quality indicators has been well studied, and significant correlations with pathogens *Giardia* spp. and *Cryptosporidium* spp. (Ferguson et al., 1996), Synthetic Organic Compound (SoC) and nitrogen–ammonia (NH_3_–N) substances (Nnane et al., 2011) and fecal indicators (Ferguson et al., 1996; Herrig et al., 2019) have been observed. In our study, metacommunity 2 included most members that were positively associated with turbidity, while metacommunity 1 displayed a mixed response and the third contained few taxa showing association to this factor. Using turbidity as a proxy, we showed that an increased abundance of metacommunity‐2 members, such as the genus *Illumatobacter* and *Nitrosospira*, in freshwater collected at important raw drinking water resources in Sweden would mimic that of a reduction in water quality and thus, a potential increase in risk for the consumers. A potential approach to implement these findings in practical use in water management and surveillance activities of important water resources would be the development of qPCR‐based markers that target these community members for rapid detection of reduced water quality (McLellan & Eren, 2014).

Of note is that metacommunity 2, shown to be positively correlated with turbidity, displayed several orthologues present in pathways involved in methane metabolism at a high likelihood of increased abundance. As methanogenic and methanotrophic processes are associated with anaerobic metabolism predominantly, this may point to the possibility of anoxic micro‐environments present in particle‐associated niches common in waters with high turbidity, or direct association of methane‐producing taxa with photoautotrophic species by direct transfer of substrates (Grossart et al., 2011).

Metacommunity 3 showed a higher abundance of orthologues associated with lipid metabolism (although this was not pronounced) as well as the highest abundance of carbohydrate metabolism‐related orthologues among the three metacommunities, potentially linking this metacommunity with nutrient‐rich waters, supported by the association of this metacommunity with elevated levels of COD‐Mn (chemical oxygen demand).

In conclusion, the findings presented here show that bacterial communities at six Swedish raw drinking water sources are subjected to selective pressure from environmental and land use conditions. Anthropogenic perturbation results in an effect on the structure and composition of the bacterial communities although the microbial taxa constituting the anthropogenic signature differ. Across this gradient, the communities were structured into three metacommunities which were present at all locations across the study period, albeit at different frequencies, and consisted of typical freshwater families such as Burkholderiaceae, Flavobacteriaceae, Commamonadeceae, and Pseudomonadaceae. Bacterial lineages within metacommunities showed strong correlation and, thus, preference for occupying the same ecological niches. Between metacommunities, lineages correlated negatively. By predicting metabolic functions of the communities, the same metacommunity structure was recovered, supporting this finding. An important goal for future research is to study competing and co‐existing bacterial lineages to better understand their role when aquatic systems are impacted by anthropogenic stress.

## AUTHOR CONTRIBUTIONS

**Björn Brindefalk**: Conceptualization (equal); data curation (equal); formal analysis (equal); investigation (equal); methodology (equal); project administration (equal); writing – original draft (equal); writing – review & editing (equal). **Harald Brolin**: Conceptualization (supporting); formal analysis (supporting). **Melle Säve‐Söderbergh**: conceptualization (equal); writing – original draft (equal); writing – review & editing (equal). **Edvin Karlsson**: Conceptualization (equal); writing – original draft (equal); writing – review & editing (equal). **David Sundell**: Conceptualization (equal); writing – original draft (equal); writing – review & editing (equal). **Per Wikström**: Conceptualization (equal); formal analysis (equal); writing – original draft (equal); writing – review & editing (equal). **Karin Jacobsson**: Conceptualization (equal); writing – original draft (equal); writing – review & editing (equal). **Jonas Toljander**: Conceptualization (equal); writing – original draft (equal); writing – review & editing (equal). **Per Stenberg**: Conceptualization (equal); methodology (equal); writing – original draft (equal); writing – review & editing (equal). **Andreas Sjödin**: Conceptualization (equal); writing – original draft (equal); writing – review & editing (equal). **Rikard Dryselius**: Conceptualization (equal); investigation (equal); writing – original draft (equal); writing – review & editing (equal). **Mats Forsman**: Conceptualization (equal); writing – original draft (equal); writing – review & editing (equal). **Jon Ahlinder**: Conceptualization (equal); data curation (equal); formal analysis (equal); investigation (equal); methodology (equal); project administration (equal); resources (equal); software (equal); supervision (equal); validation (equal); writing – original draft (equal); writing – review & editing (equal).

## CONFLICT OF INTEREST

None declared.

## ETHICS STATEMENT

None required.

## Data Availability

All sequencing data generated in Hägglund et al. (2018) is available at Short Read Archive, accession SRP159537: https://www.ncbi.nlm.nih.gov/bioproject/PRJNA489201; **Table S1** is available in the Zenodo repository at https://doi.org/10.5281/zenodo.7066483 (Table S1: All associations between ASVs and predictors included in the GLLVM).

## APPENDIX A

See Figures A1, A2, A3, A4, A5, A6, A7, A8, A9, A10, A11, A12, A13 and Tables A1, A2, A3.

**Table A1:** Model selection scores where each model included a single predictor (otherwise the same as the full model)

Predictor	*df*	AIC	BIC
NULL	800	340547.3	343022.3
Location	1998	329758.6	335939.9
Season	1598	336090.7	341034.5
Temperature	1198	337340.6	341046.9
Turbidity	1198	337194.3	340900.7
CODMn	1198	337543.9	341250.2
Color value	1198	337206.6	340912.9

*Note*: Higher scores indicate less good model performance. The NULL model did not include any predictors.

Abbreviations: AIC, Aikake information criterion score; BIC, Bayesian information criterion score; *df*, model degrees of freedom.

**Table A2:** The top significant associations of the 200 included taxa and predictors in the GLLVM analysis results

Phylum	Family	Genus	Metacommunity	Predictor	Estimate (95% CI)
Ochrophyta	Bacillariophyta	‐	3	Temperature	2.8 (1.1, 4.5)
				Color value	−7.2 (−13.2, −1.3)
				Motala site	−26.6 (−26.6, −26.6)
Bacteroidetes	Flavobacteriaceae	Flavobacterium	3	Temperature	−1.8 (−3.4, −0.1)
Cyanobacteria	GpI	‐	1	Turbidity	1.9 (0.7, 3.04)
Verrucomicrobia	Puniceicoccaceae	Cerasicoccus	1	Turbidity	−4.0 (−7.5, −0.5)
				Borås site	31.2 (25.5, 37.0)
Acidobacteria	Holophagaceae	Geothrix	2	CODMn	4.1 (2.0, 6.2)
				Östersund site	−27.2 (−27.2, −27.2)
Verrucomicrobia	Puniceicoccaceae	Cerasicoccus	1	CODMn	−4.8 (−8.6, −1.0)
				Turbidity	−4.0 (−7.5, −0.5)
				Härnösand site	34.7 (29.7, 39.6)
				Motala site	12.8 (3.6, 22.1)
				Trollhättan site	29.2 (26.4, 32.0)
	Enterobacteriaceae	Escherichia	1	Color value	5.3 (0.0, 10.6)
	Moraxellaceae	Acinetobacter	3	Summer season	25.0 (21.7, 28.3)
				Fall season	30.4 (28.7, 32.0)
				Winter season	27.4 (24.2, 30.7)
	Cryptomonadaceae	‐	2	Summer season	−7.7 (−11.9, −3.5)
Bacteroidetes	Flavobacteriaceae	Flavobacterium	3	Fall season	−9.4 (−12.5, −6.3)
Bacteroidetes	Flavobacteriaceae	Flavobacterium	3	Winter season	−16.2 (−16.2, −16.2)
Actinobacteria	‐	‐	1	Borås site	−22,1 (‐22.1, −22.1)
				Trollhättan site	−25.3 (−25.3, −25.3)
‐	‐	‐	2	Härnösand site	−30.5 (−30.5, −30.5)
Proteobacteria	Methylococcaceae	Methyloparacoccus	1	Östersund site	20.9 (12.5, 29.3)

*Note*: For the site predictors, Stockholm was set as the reference level and for the season predictors, spring was set as the reference level.

Abbreviations: CI, confidence interval; GLLVM, generalized linear latent variable model.

**Table A3:** The number of reads per sample for the complete data set and for the trimmed data set comprising just the top 200 taxa

Location	All ASVs	Top 200 ASVs	Location	All ASVs	Top 200 ASVs	Location	All ASVs	Top 200 ASVs
Boras1	127,446	97,451	Harnosand1	218,817	166,095	Motala1	116,442	86,026
Boras10	89,897	73,656	Harnosand10	98,574	81,449	Motala10	65,166	45,031
Boras11	125,329	98,216	Harnosand11	174,886	139,978	Motala11	84,678	70,849
Boras12	156,737	129,462	Harnosand13	62,881	46,331	Motala12	231,317	166,215
Boras14	95,605	74,653	Harnosand14	83,589	61,017	Motala14	166,850	107,000
Boras15	94,280	60,044	Harnosand15	241,060	166,356	Motala15	115,584	92,286
Boras16	165,774	100,507	Harnosand16	233,951	120,098	Motala16	79,339	65,955
Boras17	175,558	133,438	Harnosand17	45,498	23,559	Motala17	137,338	107,327
Boras18	252,354	184,009	Harnosand18	105,937	73,349	Motala18	98,722	76,968
Boras19	83,475	57,759	Harnosand19	154,111	105,538	Motala19	73,696	61,883
Boras2	134,971	96,911	Harnosand2	98,660	73,942	Motala2	54,920	41,916
Boras20	80,013	56,431	Harnosand20	132,649	87,363	Motala20	44,878	39,926
Boras21	129,007	92,565	Harnosand21	88,981	64,978	Motala21	51,947	45,113
Boras22	164,352	124,591	Harnosand22	106,957	77,131	Motala22	82,506	66,499
Boras23	906,88	67,019	Harnosand23	129,048	99,960	Motala23	68,685	51,519
Boras24	58,392	44,532	Harnosand24	43,550	34,810	Motala3	56,911	44,946
Boras25	55,855	45,062	Harnosand25	43,071	33,700	Motala4	433,918	374,908
Boras26	40,200	33,575	Harnosand26	57,644	47,560	Motala5	245,189	211,234
Boras27	43,755	33,492	Harnosand27	61,024	51,587	Motala6	101,362	92,499
Boras28	43,585	37,561	Harnosand28	66,870	55,571	Motala7	170,193	154,590
Boras29	39,171	33,924	Harnosand29	61,807	50,407	Motala8	158,653	142,576
Boras3	78,542	63,590	Harnosand3	71,742	49,895			
Boras30	59,638	52,547	Harnosand4	63,957	50,788			
Boras31	65,544	49,172	Harnosand5	66,797	52,384			
Boras32	60,921	51,257	Harnosand6	53,566	41,464			
Boras4	51,350	42,031	Harnosand7	286,349	213,519			
Boras5	55,915	45,818	Harnosand8	206,095	154,958			
Boras6	339,947	270,287	Harnosand9	119,191	90,302			
Boras7	136,771	119,715						
Boras8	93,113	81,127						
Boras9	291,021	246,311						

**Location**	**All ASVs**	**Top 200 ASVs**	**Location**	**All ASVs**	**Top 200 ASVs**	**Location**	**All ASVs**	**Top 200 ASVs**
Ostersund1	131,678	106,984	Stockholm1	132,202	95,315	Trollhattan1	94,223	77,452
Ostersund11	77,746	55,171	Stockholm10	162,284	141,407	Trollhattan10	177,217	145,359
Ostersund12	78,563	58,590	Stockholm11	165,049	149,971	Trollhattan12	79,889	55,327
Ostersund13	232,183	189,834	Stockholm13	137,431	108,604	Trollhattan13	97,128	70,217
Ostersund14	187,953	158,201	Stockholm14	124,610	97,998	Trollhattan14	224,233	144,340
Ostersund15	82,747	67,673	Stockholm15	237,002	181,964	Trollhattan15	239,811	152,548
Ostersund16	67,504	50,405	Stockholm16	204,238	158,371	Trollhattan16	262,527	183,110
Ostersund17	83,749	65,255	Stockholm17	247,431	199,037	Trollhattan17	62,121	27,959
Ostersund18	86,478	60,375	Stockholm18	235,972	186,626	Trollhattan18	87,063	59,462
Ostersund19	131,425	89,887	Stockholm19	222,076	187,260	Trollhattan19	108,748	72,156
Ostersund2	106,517	90,931	Stockholm2	77,061	56,178	Trollhattan2	208,235	136,007
Ostersund20	73,757	50,744	Stockholm20	88,293	66,581	Trollhattan20	154,989	115,470
Ostersund21	79,318	61,661	Stockholm21	94,750	71,435	Trollhattan21	184,100	143,613
Ostersund22	43,466	35,306	Stockholm22	91,229	65,280	Trollhattan22	115,401	54,135
Ostersund23	37,424	31,614	Stockholm23	131,093	102,347	Trollhattan23	88,802	44,702
Ostersund25	59,307	52,481	Stockholm24	172,210	135,016	Trollhattan24	93,153	49,833
Ostersund26	62,211	55,363	Stockholm25	347,433	275,912	Trollhattan25	138,124	105,817
Ostersund27	76,285	57,576	Stockholm26	95,906	79,613	Trollhattan26	88,090	71,410
Ostersund3	44,083	37,927	Stockholm27	46,612	40,852	Trollhattan27	76,697	63,918
Ostersund4	53,049	45,565	Stockholm28	47,774	41,442	Trollhattan28	62,873	54,541
Ostersund5	376,939	323,937	Stockholm3	252,377	185,107	Trollhattan29	59,623	50,757
Ostersund6	166,074	126,610	Stockholm30	72,045	64,806	Trollhattan3	56,434	47,801
Ostersund7	124,504	109,742	Stockholm31	72,392	61,501	Trollhattan30	56,457	47,424
Ostersund8	142,539	120,941	Stockholm32	76,234	65,839	Trollhattan31	58,833	46,168
Ostersund9	187,572	164,837	Stockholm4	77,220	61,270	Trollhattan32	59,719	47,499
			Stockholm5	66,324	54,921	Trollhattan4	78,559	67,981
			Stockholm6	46,874	40,286	Trollhattan5	27,958	24,359
			Stockholm7	348,748	295,004	Trollhattan6	395,119	324,354
			Stockholm8	309,971	256,771	Trollhattan7	337,355	290,630
			Stockholm9	177,053	156,809	Trollhattan8	177,052	156,176
						Trollhattan9	129,354	111,398

Abbreviation: ASV, amplicon sequence variant.

**Materials and Methods**

**GLLVM analysis**

This model, Equation (1) in the main article, allowed us to estimate correlations between ASVs while simultaneously accounting for predictor variables. Three latent variables were used in the analysis, which is believed to capture most of the relevant variation in composition: see example in Warton et al. (2015) and Niku et al. (2019). To improve convergence, jittering of latent variables was set to 0.5 (jitter.var = 0.5). The negative binomial distribution was selected to model the count data. For further details of the modeling, please consult Niku et al. (2017, 2019). To visualize the inferred regression parameters, we made use of the ggplot2 R package.

To reduce the dimension of the co‐occurrence data, the top 200 ASVs were selected to be included in the analysis to avoid spurious correlations with low‐abundant features close to the noise level. In the analysis, we first checked whether including predictors (i.e., full model as in Equation 1 in the main paper) improved the results in terms of accounting for a large proportion of the total variation in ASV abundance as compared to a model without the predictors (i.e., null model). By including all predictors in the model, 41% of the total variation was accounted for in the analysis, which better allows us to draw conclusions from the inferred ASV correlations after adjusting for the predictors (Figure A3). For example, little or no side effects remain after including the corresponding site predictor in the model (i.e., no visible pattern in communities shown in Figure A6a), contrary to what was evident in the null model and the standard ordination results (Figure A6b and Figure).

To check for collinearity, we calculated the pair‐wise Pearson correlations between predictors (Figure A11). One out of six estimated pair‐wise correlations was large (*r* = 0.87) suggesting a potential problem of collinearity for CODMn and Color value. All other obtained estimates were between −0.1 and 0.3. Thus, they cannot independently predict the value of the dependent variable: they explain partly the same variance in the dependent variable. Because of this, caution is needed when interpreting individual associations of CODMn and color value with ASVs.

**Results**

**GLLVM analysis**

In the GLLVM analysis, associations were also determined between the bacterial assemblages and environmental, design, and water quality variables: all non‐zero associations are highlighted in Figures 5 and A7, A8, A9. Turbidity and air temperature resulted in the most significant associations. Of the taxa showing a positive response to the CODMn predictor, the majority belonged to community 1. Intriguingly, a few representatives of community 2 (consisting of members of the *Flavobacterium* genus), also displayed a positive response. Most ASVs showed a negative response to higher color values, except for two representatives from community 1 (assigned as Alkaligenaceae and Polynucleobacter). For metacommunities 1 and 2, the air temperature had a clear positive effect, with almost all taxa showing increases. A clear pattern was evident, where a significant number of taxa belonging to metacommunity 2 showed a positive response to turbidity (*n* = 26 ASVs in total), amongst them *Nitrosospira* and taxa belonging to Verrucomicrobia clade, along with a number of taxonomically undefined taxa including some belonging to metacommunity 1. Furthermore, a clear signal from cyanobacteria Family 1 corresponds to a small selection of samples where a clear cyanobacterial bloom was taking place. This result indicates that metacommunity 2 constitutes microorganisms adapted to waters with a high number of suspended particles present and could be seen, at the most basic level, as a proxy for a trophy or primary productivity. Furthermore, a number of taxa belonging to metacommunity 1 (*Pseudomonas, Comamonadaceae, Polynucleobacter, Methylococcaceae, Cerasicoccus*, and *Plancomycetaeae)* showed a negative response to higher turbidity, in line with the hypothesis that these represent more generalist species and are not well adapted to the specific circumstances found in particle rich waters, where conditions would favor more specialized organisms.

For the season predictor, most ASVs with a significant association between spring and any other season were overrepresented in the spring assemblages (i.e., a negative effect size value in Figure 4a). When contrasting summer against spring factor levels, a greater number of taxa belonging to metacommunities 1 and 2 were showing a moderate decrease during summer, while metacommunity 3 taxa showed an overrepresentation during summer. The overall autumn season response was similar to the summer response, with a few notable exceptions. Overrepresented taxa in the autumn are dominated by members of metacommunity 3, although genus *Flavobacterium* displayed a mixed response, with some taxa (*n* = 13) showing an overrepresentation and a number displaying a more pronounced underrepresentation (*n* = 12). metacommunities 1 and 3 showed a large number of taxa with a weak underrepresentation in abundance. When contrasting spring and winter factor levels, most taxa showed an underrepresentation in the winter collected assemblages (Figure 5). Of the taxa showing a larger underrepresentation, all were members of metacommunity 3 (Flavobacterium). Intriguingly two ASVs, both methanotrophs assigned to metacommunity 1, showed the largest overrepresentation in abundance across all seasons. In addition, one ASV assigned to the genus Cerasicoccus (phylum Verrucomicrobia) was overrepresented in both, the summer and autumn seasons. Furthermore, one ASV assigned to metacommunity 3 belonging to the genus Acinetobacter was also overrepresented in abundance across the seasons compared to spring.

Investigations of site effects on ASVs revealed that generally, ASVs assigned to metacommunities 1 and 2 showed a higher number of associations than ASVs assigned to metacommunity 3, further reinforcing that metacommunity three members consist of generalist bacteria (Figure 5b). A set of ASVs showed similar associations across multiple sites with Verrucomicrobia, genus Cerasicoccus, being overrepresented in terms of abundance at the Borås, Trollhättan, and Härnösand sites. One ASV classified as methanotroph (*Methyloparacoccus*) was overrepresented in Östersund and Härnösand sites. One ASV assigned as a member of the *Pedobacter* genus (Bacteroidetes) was overrepresented in Stockholm compared to both Härnösand and Motala, while one ASV without any assignment at the phylum level, placed in the Illumatobacter clade was overrepresented in Stockholm compared to Östersund and Motala. When compared to Stockholm, Härnösand showed the greatest divergence in response of taxa, with 39 and 51 ASVs out of 200 significantly over‐ and underrepresented in Härnösand, respectively. The water in Trollhättan is sourced from a river and thus subject to varying conditions in the upstream sources, which manifested as a large portion (*n* = 102) of the 200 ASVs being associated with a site difference, mostly overrepresented in the Stockholm assemblages.

**Variable importance analysis**

One goal was to determine the importance of the included predictors in the GLLVM analysis, both in terms of the goodness‐of‐fit of the model to the raw water assemblage data, and model complexity (i.e., more complex predictors require more degrees of freedom in the model). To do so, separate models with a single predictor were fitted to the data, with AIC and BIC scores calculated by the GLLVM R package: low scores indicated a better‐suited model. All models were analyzed using the same parameter set as the full model analysis. The most important predictor by far was the location of the raw water sources, even though this predictor required the highest degrees of freedom, suggesting that the gradient across water sources induced the highest variation in composition (Table S1) and accounted for approximately 20% of the total variation. The other set of predictors resulted in very similar model performance with 0%–5% of the total variation explained, and thus much less variation explained than the location predictor. Following AIC, the season effect was the second most important predictor, while BIC preferred turbidity (BIC penalized degrees of freedom more than AIC). However, all predictors resulted in marginally better models than the null model without predictors. Color value and turbidity resulted in very similar model performance.
